# The Use of Prone Magnetic Resonance Imaging to Rule Out Tethered Cord in Patients With Structural Spine Anomalies: A Diagnostic Technical Note for Surgical Decision-making

**DOI:** 10.7759/cureus.4221

**Published:** 2019-03-11

**Authors:** Salah G Aoun, Tarek Y El Ahmadieh, Awais Z Vance, Om Neeley, Kevin C Morrill

**Affiliations:** 1 Neurosurgery, University of Texas Southwestern Medical Center, Dallas, USA

**Keywords:** tethered cord syndrome, prone magnetic resonance imaging, fatty filum terminale, structural spinal cord abnormality, anterior conus movement

## Abstract

Tethered cord syndrome (TCS) is a clinical diagnosis that can be difficult to establish, as symptoms do not always match classic radiological findings, such as a low-lying conus. Surgery for spinal detethering is not without risk and does not always result in clinical improvement. Prone magnetic resonance imaging (MRI) has been described as a tool to assess the mobility of the spine. This is a technical imaging report where prone imaging was a factor that influenced the decision to defer surgery in favor of conservative management. T1 and T2 sagittal and T1 axial MRI imaging were obtained with the patient supine, and then repeated in the prone position. An anteroposterior conus movement of >10% of the canal width was considered normal. There was significant anterior movement of the conus when switching to the prone position. Surgery was deferred, and the patient improved after a regimen of intensive physical therapy. Prone MRI can be a useful tool to have in our neurosurgical armamentarium when assessing spinal cord tethering. Surgery is not recommended when normal anteroposterior movement of the conus is present.

## Introduction

The diagnosis of tethered cord syndrome (TCS) can be challenging to establish [1], and the surgical solution is not without risk and does not always guarantee clinical improvement [2]. Prior studies have shown that standard magnetic resonance imaging (MRI) criteria such as a low-lying conus terminalis, or the presence of a fatty filum have a low imaging sensitivity and specificity, and thus cannot be used reliably to make a diagnostic correlation with clinical symptoms [3-5]. The surgical procedure itself can be of variable complexity and range from simple filum sectioning, to a more tenuous dissection in cases of re-tethering such as in functional spinal bifida patients [6-7]. While surgery can provide significant symptomatic improvement in the right patient, complications are not benign and range from spinal fluid leakage and meningitis, to wound dehiscence requiring expansile flap grafting, with a chance of persistence or early recurrence of the presenting neurological symptoms [8-9]. There have been reports of using prone MRI to assess the anteroposterior movement of the conus in the setting of TCS, but most studies have involved pediatric populations, or have been retrospective and purely descriptive in nature, and provided equivocal results [3-4]. We present a case where comparative imaging between prone and supine MRI was used to successfully steer the decision towards the non-surgical management of a patient with split cord malformation and low-lying conus, who presented with equivocal symptoms.

## Case presentation

**Figure 1:**
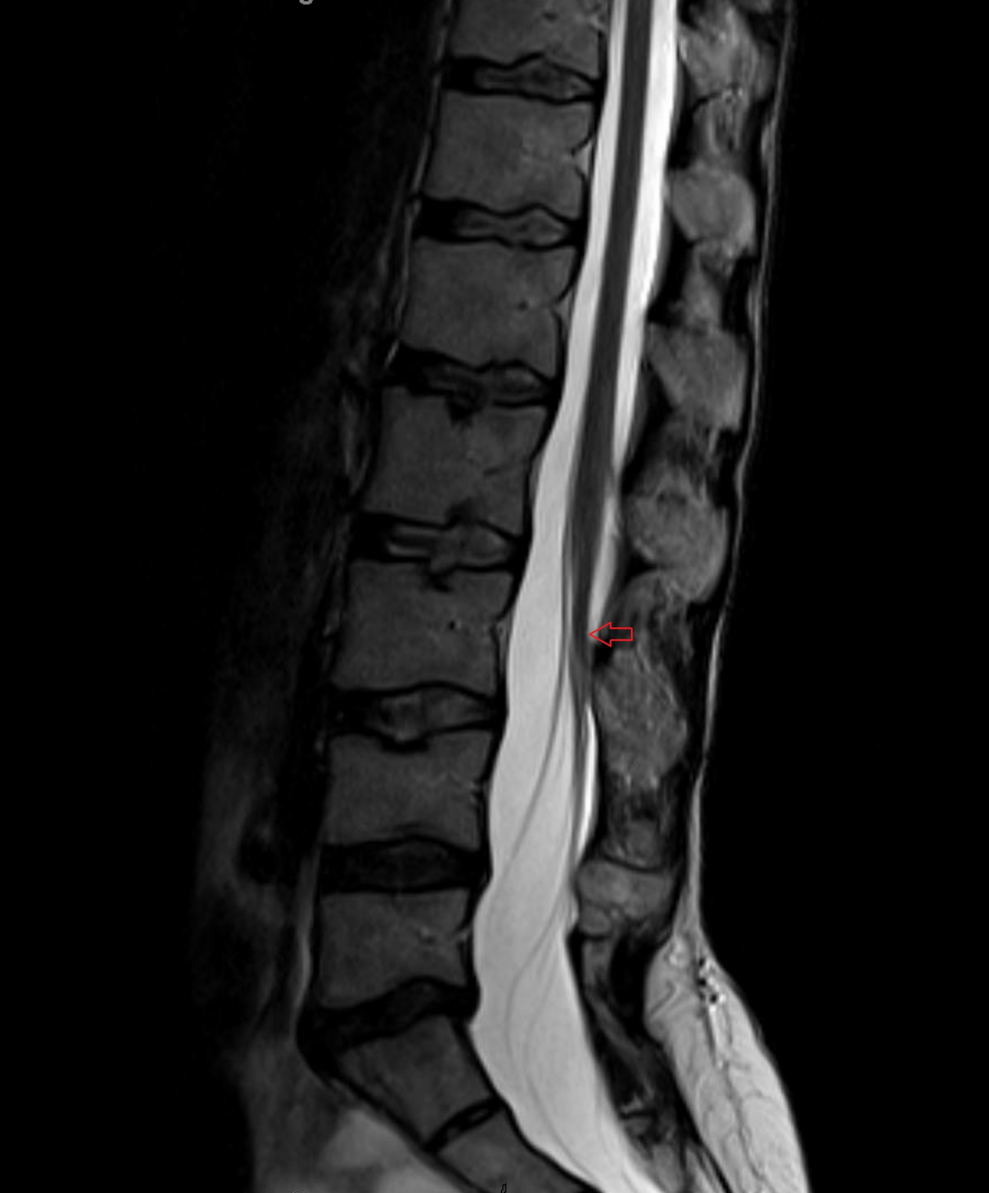
Sagittal T2 magnetic resonance imaging scan in the supine position showing a low-lying conus at the level of the mid-body of the L3 vertebral body (red arrow)

A 50-year-old woman presented to our clinic with complaints of worsening mechanical axial lower back pain for the past 10 years that had now become debilitating, and intermittent bilateral radicular components towards the end of the day that were poorly defined. She worked as a nurse, and had a history of a small patch of hair that was removed from her mid lower back when she was four years old, without reported surgical exploration. She carried the diagnosis of tethered cord syndrome. Her clinical examination was benign except for mild diffuse hyperreflexia. A standard 3 Tesla MRI of the lumbar spine was obtained in the supine position and included axial T1 and T2 cuts, as well as T2 sagittal reconstructions. The T2 sagittal reconstructions were used to assess the position and the motion of the conus, and the axial T1 images were used to assess the presence of a fat-infiltrated filum. We then positioned the patient prone and obtained the same sequences. Normal ventral motion of the conus was defined as >10% of the total antero-posterior canal width as previously described by Stamates et al. [3]. Her supine MRI showed a low-lying conus medullaris at the level of the L3-4 disc space (Figure 1).

**Figure 2:**
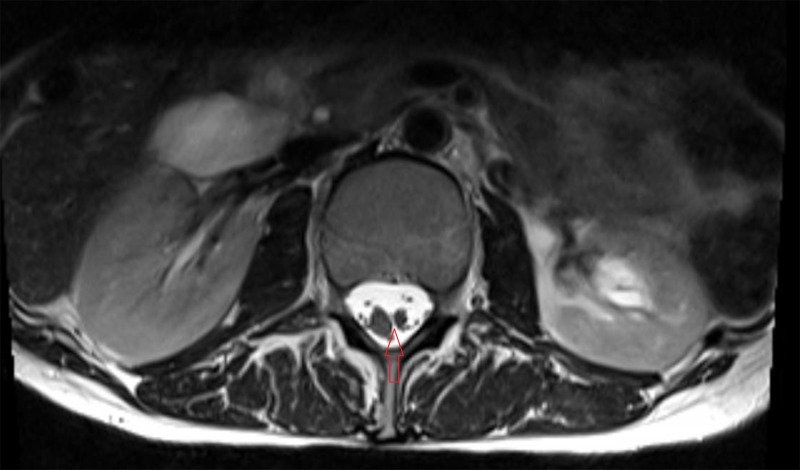
Axial T2 magnetic resonance imaging scan showing a split cord malformation (red arrow)

Her axial images showed a split cord malformation, without the presence of a bony septation (Figure 2).

**Figure 3:**
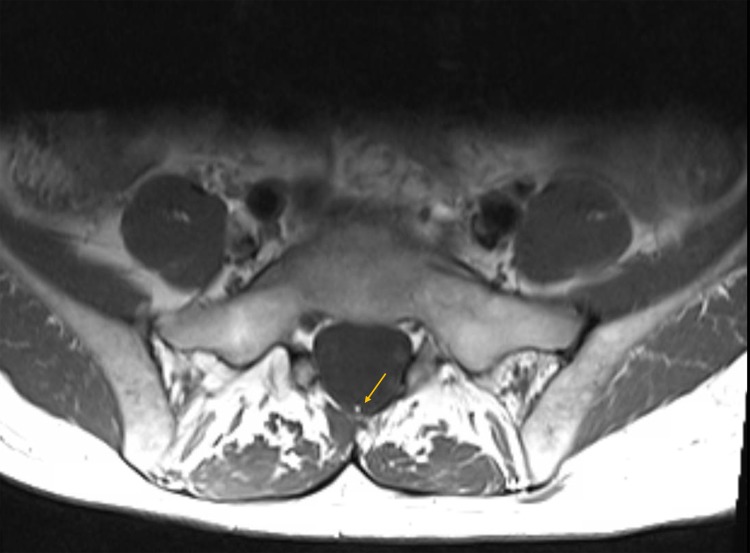
Axial T1 magnetic resonance imaging scan at the level of the L5 vertebral body showing a fatty filum (yellow arrow)

Axial imaging at the level of L5-S1 revealed a small fatty filum terminale (Figure 3).

**Figure 4:**
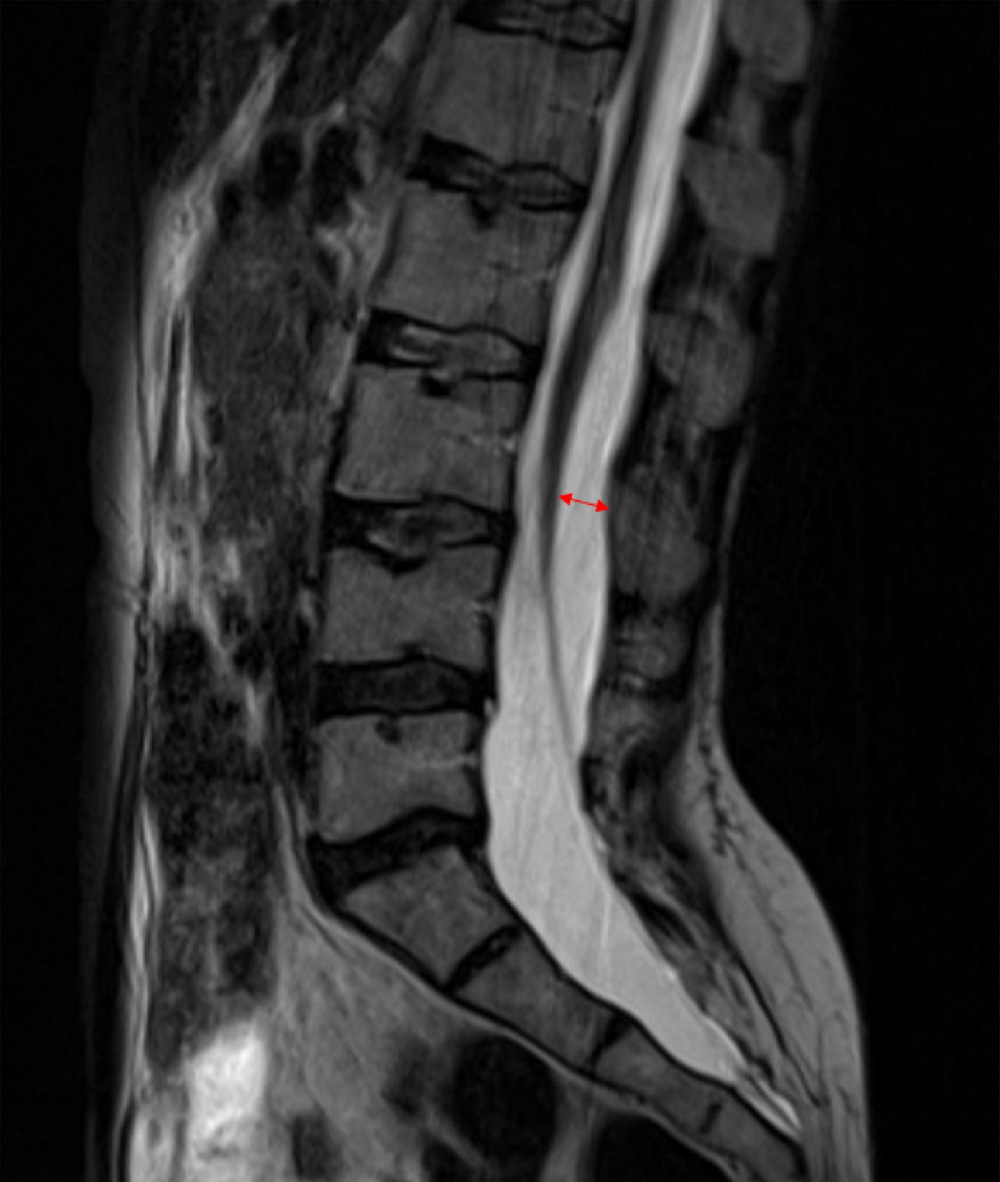
Sagittal T2 magnetic resonance imaging scan in the prone position showing adequate anterior movement of the conus compared to the supine imaging (red double arrow)

A prone MRI was obtained (Figure 4).

It showed significant anterior motion of the spinal cord of more than 10% of the central canal width when comparing prone to supine sagittal T2 images. She was prescribed an intensive physical therapy regimen for 12 weeks, and her symptoms completely resolved but her hyperreflexia persisted.

## Discussion

Assessing conus terminalis anterior movement can be a useful way to rule out spinal cord tethering in adult patients showing anatomical features of structural cord malformation, such as spina bifida, split cord malformation, and a fatty filum terminale. The usefulness of obtaining a prone MRI has been previously described, but mostly in pediatric patients series, and in a retrospective fashion after surgery had already been performed [3-4,10-11]. Although this report is based on a single case, it showcases the practical advantage of obtaining prone MRI imaging in adult patients presenting with atypical spinal complaints such as back pain with ambulation and intermittent radicular symptoms, especially if they have an associated anatomical spinal cord anomaly. The prone sagittal MRI reconstructions can count as an additional argument towards managing lower back complaints conservatively and deferring surgery, if the conus appears to have normal anteroposterior motility. In their descriptive retrospective study of 41 patients with tethered cord malformation, Stamates et al. determined that an anteroposterior movement greater than 10% of the spinal canal width when comparing prone and supine imaging, was both sensitive and specific to rule out tethering [3]. Their review was retrospective, and their data was obtained by comparing tethered patients to normal controls, but their concept still holds true when assessing potential surgical candidates prospectively.

## Conclusions

Prone MRI can be a useful tool in the armamentarium of neurosurgeons when assessing adult patients with ambiguous clinical and imaging findings of spinal cord tethering, especially if they appear unlikely to benefit from surgical untethering. We hope that this report will be helpful to clinicians by providing them with an additional diagnostic option and treatment algorithm when considering these patients for surgery.
